# The Role of MEF2 Transcription Factor Family in Neuronal Survival and Degeneration

**DOI:** 10.3390/ijms24043120

**Published:** 2023-02-04

**Authors:** Malwina Lisek, Oskar Przybyszewski, Ludmila Zylinska, Feng Guo, Tomasz Boczek

**Affiliations:** 1 Department of Molecular Neurochemistry, Medical University of Lodz, 92-215 Lodz, Poland; 2 Department of Pharmaceutical Toxicology, China Medical University, Shenyang 110122, China

**Keywords:** myocyte enhancer factor 2, neuronal survival, neurodegeneration, neuropsychiatric diseases, transcription factors

## Abstract

The family of myocyte enhancer factor 2 (MEF2) transcription factors comprises four highly conserved members that play an important role in the nervous system. They appear in precisely defined time frames in the developing brain to turn on and turn off genes affecting growth, pruning and survival of neurons. MEF2s are known to dictate neuronal development, synaptic plasticity and restrict the number of synapses in the hippocampus, thus affecting learning and memory formation. In primary neurons, negative regulation of MEF2 activity by external stimuli or stress conditions is known to induce apoptosis, albeit the pro or antiapoptotic action of MEF2 depends on the neuronal maturation stage. By contrast, enhancement of MEF2 transcriptional activity protects neurons from apoptotic death both in vitro and in preclinical models of neurodegenerative diseases. A growing body of evidence places this transcription factor in the center of many neuropathologies associated with age-dependent neuronal dysfunctions or gradual but irreversible neuron loss. In this work, we discuss how the altered function of MEF2s during development and in adulthood affecting neuronal survival may be linked to neuropsychiatric disorders.

## 1. Introduction

MEF2 (myocyte enhancer factor 2) was originally identified in 1989 as a nuclear factor interacting with the creatine kinase gene enhancer during muscle differentiation [1]. It turned out quickly that it is ubiquitously expressed in a variety of tissues, including the brain, and its function exceeds far beyond muscle physiology [2]. The transcription factors of the MEF2 family are highly evolutionary conserved and play a pivotal role in a variety of vital processes including survival, differentiation and response to extracellular stimuli. In the nervous system, MEF2 acts as a central player in neuronal survival and axonal outgrowth by controlling the expression of numerous genes and miRNAs [3,4,5]. Its role has been documented both during brain development and in mature neurons. For instance, activation of MEF2 reduces the number of dendritic spines and restricts the excitatory synapse number by promoting degradation of the scaffold protein, postsynaptic density protein 95 (PSD95) [6,7]. Conversely, several studies demonstrated that inhibition of MEF2 activity through knockdown or gene deletion increased the number of synapses [8,9,10] and affected cognitive functions [11]. MEF2 isoforms regulate basal synaptic transmission, which involves neurotransmitter release at individual synapses, as well as evoked transmission involving action potential-induced fusion of synaptic vesicles. Both types of synaptic transmission are important for hippocampal-dependent learning and memory formation [9,12], as well as development of molecular pathologies in some neuropsychiatric diseases [13,14,15]. In this context, activity-dependent MEF2 action should be considered as a mechanism by which sensory-motor experience regulates gene expression and affects synaptic function early in brain development and through adulthood.

A recent study reported that MEF2 transcription factors are implicated in long-term synaptic depression, which is thought to initiate synapse elimination and may lead to cerebellar dysfunctions [16]. It has also been reported that Lys-403, the sumoylated form of MEF2A, promotes the formation of dendritic claws in the cerebellar cortex, while its acetylated form inhibits the differentiation of postsynaptic granule neurons [17]. A growing body of evidence clearly indicates that MEF2 has a role in brain organogenesis and suggests its contribution to neuronal deficits [2,18]. Therefore, in this review we focus on the relationship between the MEF2 family of transcription factors and the mechanisms of neuropsychiatric diseases at the cellular and molecular level.

## 2. MEF2 Transcription Factors—Structure and Regulation

**Figure 1:**
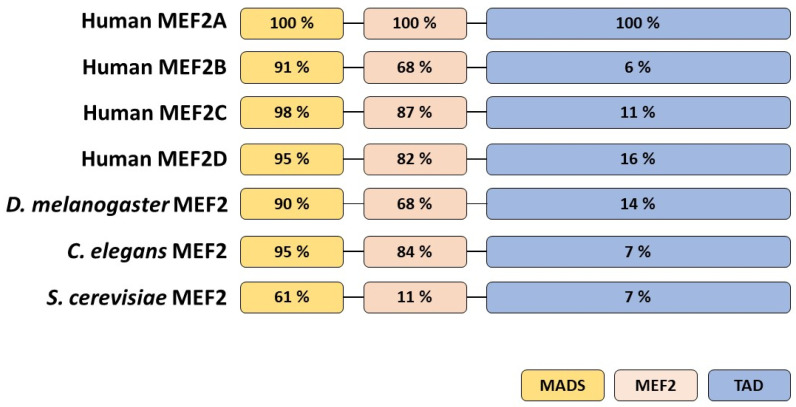
Domain homology of the MEF2 transcription factors family across different organisms. The percentage of sequence identity in relation to human MEF2A taken as 100%. The N-terminus is on the left. MADS—MCM1, AGAMOUS, DEFICIENS and SRF, serum response factor domain; MEF2—myocyte enhancer factor 2 domain; TAD—topologically associated domain. Adapted from [32].

In vertebrates, four MEF2 isoforms encoded by distinct genes named MEF2A, MEF2B, MEF2C and MEF2D have been identified, whereas a single MEF2 gene has been reported in *D. melanogaster*, *C. elegans* and *S. cerevisiae* [19] (Figure 1). Gene mapping in mice showed that MEF2A, MEF2B, MEF2C and MEF2D are located at chromosome 7, 8, 13 and 3, respectively [20]. In humans, MEF2A, B, C and D genes have been mapped at chromosomal locations 15q26, 19p12, 5q14 and 1q12-q23, respectively [21,22]. Phylogenetic analysis of the MEF2 gene’s origin, conservation and evolution [23] demonstrated that MEF2B is clearly distant from the other three MEF2s in vertebrates and its origin is presumably the most ancient. MEF2A and MEF2C share a high similarity as they both result from a duplication event occurring near the origin of the vertebrate. Consistent with the structural data, MEF2A, MEF2C and MEF2D (but not MEF2B) contain the HJURP_C region that appeared in evolution later than N-terminal conserved motif, the MADS-box/MEF2s [24] domain, suggesting that all three paralogues may have divergent from a single ancestor. This similarity is also reflected by some common types of alternative splicing in MEF2A and MEF2C transcripts [19].

The MEF2 family belongs to the type II MADS domain-containing proteins (Minichromosome Maintenance 1, Agamous, Deficiens and Serum Response Factor). In contrast to type I proteins having the SAM (SRF, ARG80, MCM1) domain, the type II family is characterized by the presence of the MEF2 domain as a second conserved domain. The MADS domain is responsible for binding to the CC[A/T]_6_GG motif in DNA, whereas the MEF2 domain regulates DNA-binding affinity and interactions with cofactors and other MEF2 proteins [25], although neither the MADS-box nor the MEF2 domain has its own transcriptional activity. The C-terminal transactivation domain is highly divergent among MEF2s due to tissue-specific alternative splicing [19]. This diversity allows MEF2s to interact with various transcriptional regulators, including co-activators, such as p300 and CBP (CREB-binding protein) acetyltransferases [26,27,28] or co-repressors, such as class II histone deacetylases and the nuclear receptor co-repressor/SMRT (silencing mediator of retinoic acid and thyroid hormone receptor) co-repressor [29,30,31].

As MEF2s have been found to interact with both histone acetyltransferases (HATs) and histone deacetylases (HDACs), a model describing dual MEF2 function was proposed by McKinsey and coworkers in the early 2000s [33]. According to this model, MEF2 associates with class IIa HDACs in the resting state and co-recruits class I HDACs to specific loci of the genome to inhibit the transcription of target genes. Various upstream signals, for instance differentiation in myocytes or activation of T cell receptors in thymocytes, lead to dissociation of HDACs from MEF2 via Ca^2+^-dependent mechanisms. This allows DNA-bound MEF2 to interact with Ca^2+^-sensitive transcription factors, such as NFAT or CREB, and recruits p300/CBP for transcriptional activation [34,35]. The interaction of HDAC and p300/CBP can regulate MEF2-dependent transcription directly by controlling the acetylation state of MEF2 [36,37]. The overall MEF2-regulated transcription state is tightly controlled by the mutually exclusive effect of HDAC or p300/CBP binding that constitutes the major mechanism by which MEF2 influences the activation or repression of a regulatory element.

**Figure 2:**
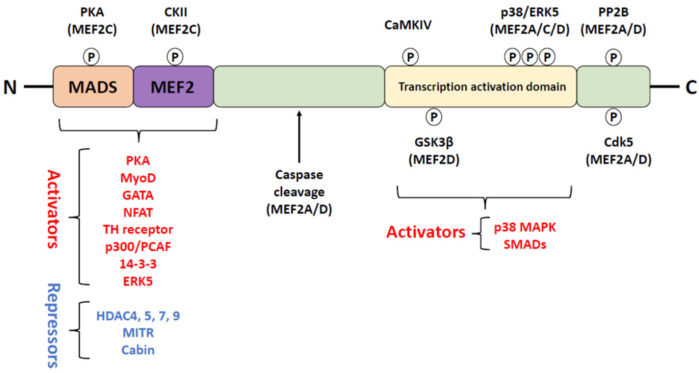
MEF2 protein interactions and the mechanisms of activity regulation. MEF2 transcription factors contain MADS at their N-terminus that mediates homo and heteromerization and DNA binding. Adjacent to MADS is the MEF2 domain that regulates cofactor binding and determines DNA-binding affinity. Several MEF2-interacting partners have been identified. They may either stimulate (activators, red color) or repress MEF2 activity (repressors, blue color). PKA (protein kinase A), CKII (casein kinase II), CaMKIV (Ca^2+^/CaM-dependent protein kinase type IV), p38 kinase, ERK5 (extracellular signal-regulated kinase type 5) and PP2B (protein phosphatase type 2B) increase MEF2 activity, whereas GSK3β (glycogen synthase kinase 3β), cdk5 (cyclin-dependent kinase type 5) and caspase cleavage inhibit MEF2 activity. Adapted from [33].

Another mechanism of MEF2A activity regulation is phosphorylation/dephosphorylation that can affect its DNA-binding affinity and interaction with transcriptional co-activators and co-repressors [2]. Several phosphorylation sites in the MEF2 transactivation domain have been identified representing the diverse regulation by various kinases and phosphatases (Figure 2).

For instance, phosphorylation by protein kinase A and casein kinase II outside of the transactivation domain increased MEF2 binding to DNA [38]. Ser408/Ser444 phosphorylation of MEF2A/D by cyclin-dependent kinase 5 (Cdk5) reduced MEF2 transcriptional activity in primary cortical neurons and mediated Cdk5-dependent neuronal apoptosis [39]. The underlying mechanism is not completely understood, but it has been suggested that Ser444 phosphorylation increases MEF2 susceptibility to protease cleavage/degradation pathways [40]. Contrary to Cdk5-induced inhibition, MEF2 phosphorylation by p38 MAP kinase or ERK5 at sites distinct from Ser444 activated MEF2 promoting neuronal survival [39,41,42]. Interestingly, Cdk5-dependent phosphorylation also increased the Lys403/Lys439 sumoylation of MEF2A making it a transcriptional repressor [17].

The transcriptional activity of MEF2 can also be modulated directly or indirectly in a Ca^2+^- and calmodulin (CaM)-dependent manner. Phosphorylation by Ca^2+^/CaM-dependent protein kinase type IV (CaMKIV) increased MEF2D transcriptional activity in T lymphocytes in vitro [43]. In neurons, however, the MEF2-regulated transcription pivotal for synaptic plasticity and survival seems to be driven by CaMKII [44]. In cerebellar granule neurons, depolarization-induced Ca^2+^ influx activated CaMKII, which phosphorylated HDAC5 to inhibit it. Phosphorylated HDAC5 dissociates from MEF2 and is exported from the nucleus with the assistance of the 14-3-3 protein [45]. This results in sustained MEF2 activity, which is free to bind transcriptional coactivators and initiate the expression of prosurvival genes.

Dephosphorylation is another key regulatory switch for MEF2 activity. Among downstream Ca^2+^/CaM-dependent targets, calcineurin (phosphatase 2B, PP2B) is implicated in competitive regulation of a variety of neuronal functions including, synaptic transmission, gene expression, memory and learning [46,47,48]. In hippocampal neurons, calcineurin-dependent dephosphorylation of MEF2 in response to increased neuronal activity suppressed the excitatory synapse number, which may negatively affect memory formation [6]. Calcineurin-regulated maintenance of MEF2 in a hypophosphorylated state has also been demonstrated to maximize its transactivation capacity and DNA binding, both critical for calcium-dependent survival of cerebellar granule cells [49]. Shalizi and colleagues showed that the transcriptional repressor form of Lys403 sumoylated MEF2A positively regulated the morphogenesis of postsynaptic granule neurons in the cerebellar cortex [17]. Activity-dependent calcium and calcineurin signaling promotes MEF2A dephosphorylation at Ser408 and a switch from sumoylation to acetylation at Lys403, which restricts dendritic claw differentiation. However, regulation of MEF2 by calcineurin and its plausible physiological consequences seem to be isoform specific. For instance, MEF2C transcriptional activity appears to be regulated in a unique manner, as Ser412 dephosphorylation by calcineurin was accompanied by MEF2C nuclear translocation. The underlying mechanism involves calreticulin working upstream of calcineurin and Ca^2+^-dependent signaling linking the endoplasmic reticulum to the nucleus [50]. Interestingly, calreticulin expression is regulated by MEF2C both in vivo and in vitro [50]. In the nucleus, MEF2 may interact with the catalytic subunit of protein phosphatase 1α (PP1α), and this interaction is thought to repress MEF2-dependent transcription [51]. Moreover, MEF2-PP1α recruits HDAC4 to the complex and prolongs its nuclear retention. Hence, repression of MEF2 activity by PP1α may withstands the effects of calcineurin signaling and alters the prosurvival MEF2 function in hippocampal neurons [51].

Besides the mechanisms discussed above, MEF2 activity may also be regulated by transcriptional and translational control of the protein level [52] as well as chaperone-mediated autophagy [53]. Altered MEF2 protein levels were found in mouse models of Parkinson’s disease [54,55] and a PS19 model of tauopathy [12] further highlighting the multifactorial and complex regulation of MEF2 activity in the brain.

## 3. The Expression of MEF2 in the Brain

The expression of four MEF2 isoforms has been detected in neonatal and adult brain regions critical for basic neuronal processes, such as information processing, memory formation or motor coordination [2,12,56,57]. Different MEF2 subtypes often present an overlapping expression pattern, but they also appear to be uniquely regulated depending on the brain region or cell type.

The correlation between MEF2 expression and morphogenetic events during brain development is especially striking in the cerebellum. The presence of all four MEF2 genes has been demonstrated in the external granule layer at birth. MEF2A and MEF2D are continuously expressed in the granule cells in the adulthood and are required for normal circadian and sleep behavior [58]. In contrast, the expression of MEF2B decreases during development and is not detected in mature granular, molecular or Purkinje cells. The expression of MEF2C can be already seen between E11 and E13, and by E17 it is predominant in the primordium of the granule layer of the cerebellar cortex [59]. This correlates well with the migration pattern of Purkinje neurons. In comparison to other members of the MEF2 family, the expression of MEF2C in the postnatal brain is restricted to only these types of neurons. Interestingly, the loss of MEF2C expression did not affect the growth and migration of Purkinje cells but altered their dendritic arborization and increased the number of glutamate decarboxylase (Gad67) and vesicular glutamate transporter (vGluT1) puncta [60].

During hippocampal development the pattern of MEF2 expression correlates well with the main time frames of neuronal differentiation. In the embryonic stage, MEF2 transcripts demonstrate an overlapping expression pattern, which becomes distinct postnatally. This switch is especially visible in the Horn and Ammon cells [56]. The MEF2B gene transcripts decrease during hippocampal maturation along with the punctate expression of MEF2C suggesting their gradual silencing in a certain population of neurons or the activation of programmed cell death. Interestingly, the data derived from MEF2A/C/D triple knockout suggest that MEF2C is the main isoform involved in hippocampal function and MEF2A and MEF2D have only subtle roles [61]. MEF2C is also the first of four MEF2 transcripts detected during cortical development and is highly expressed in immature cortical excitatory neurons [62]. The newest findings indicate that the temporal control of MEF2C expression is necessary to influence gene expression affecting multiple types of cortical excitatory neurons [63]. The presence of MEF2C mRNA can be detected in the intermediate zone of the frontal cortex as early as at E12.5 in mice. The increase in MEF2C expression is further observed during the migration of differentiated cells into the cortex and the formation of six distinct cortical layers. Early research with [^3^H] thymidine injections showed the presence of MEF2B mRNA in these six layers of neurons but MEF2C was predominantly in layers II, IV and VI of a mature neocortex [64,65]. This restricted pattern of expression in the cortex suggests that the MEF2C transcription factor may play a pivotal role in the development of cortical architecture and function, which is supported by several recent studies [62,66,67].

The developing brain, frontal cortex and thalamus are the regions containing differentiating neurons. The intensive differentiation in the ventral thalamus can be seen as early as E10, but the process continues through E16 in mice. The transverse and coronal sections of embryo hybridized with cRNA probes showed the predominance of MEF2A in the developing thalamus [56]. The signal intensity was higher for lateral neurons compared to the medial cells. The parasagittal sectioning of E16 of the mouse brain demonstrated a higher density of the signal in the caudal thalamus when compared to rostral region. These, and other data [68,69], suggest an important role of MEF2A in thalamus function development. The expression patterns of MEF2 genes in the brain at different stages of development are summarized in Table 1.

## 4. MEF2 in Neuronal Survival

Early studies on primary neuronal cultures demonstrated that MEF2 transcriptional activity is necessary to regulate neuronal survival. In postmitotic cerebral cortical neurons where MEF2C was selectively expressed, introduction of a dominant MEF2 form induced apoptosis. Similarly, overexpression of a dominant-interfering MEF2 decreased the survival of cerebellar granule cells cultured in depolarizing conditions [70]. On the other hand, the survival of granule neurons expressing the constitutively active form of MEF2 was enhanced in the absence of external depolarizing stimuli, the condition that is expected to promote apoptosis [70]. Another line of evidence of the prosurvival role of MEF2 comes from RNA interference experiments that showed a reduced survival of granule cells when MEF2A expression was silenced [71]. It is known that MEF2 transcription factors are activated by p38 mitogen-activated kinase and the MEF2-p38 pathway is antiapoptotic during development but proapoptotic in mature neurons exposed to stress insults [41,72]. In line with that, Okamoto and colleagues demonstrated that activation of the N-methyl-D-aspartate (NMDA) receptor in mature cerebrocortical neurons resulted in the caspase-mediated cleavage of MEF2A, C and D. The cleavage products containing the transactivation domain, but lacking DNA binding ability, blocked MEF2 transcriptional activity via dominant interference, ultimately leading to apoptosis [40]. Overexpression of the constitutively active mutant of MEF2C rescued MEF2 transcriptional activity and preserved neuronal survival. Inhibition of NMDA-mediated neuronal apoptosis was also seen when the p38-MEF2 pathway [72,73] was blocked, further highlighting a dualistic nature of this pathway as either pro or antiapoptotic. Although p38 MAP kinase phosphorylation of MEF2 is implicated in the Ca^2+^-dependent regulation of MEF2 activity, the downstream targets underlying the prosurvival role of MEF2 remain unknown. Of paramount importance are also the upstream regulators of neuronal cell death, especially electrical activity necessary for the survival of young neurons, that affect Ca^2+^ homeostasis and confer proper activation of Ca^2+^ cascades including the activation of MEF2 [74]. 

In vitro transfection of stem-like P19 cells or murine embryonic stem cells with a constitutively active form of MEF2C was antiapoptotic and induced a mixed neuronal/myogenic phenotype [41]. However, forced expression of a dominant negative form of MEF2C prevented retinoic acid-dependent differentiation and enhanced apoptosis without interfering with cell proliferation. The dominant negative form of p38 kinase also increased neuronal mortality, but the survival could be rescued by overexpressing constitutively active MEF2C. In P19 cells, this MEF2 isoform was able to induce neuronal differentiation and promoted the expression of neuronal proteins interacting with MEF2s [75]. This data indicates that MEF2 may not only determine cell survival under certain conditions but regulate differentiation and neurogenesis.

Neuronal stem cells (NSCs) are self-renewing multipotential stem cells that can proliferate in vitro and differentiate into neuronal and/or glial lines. NSCs for neural cell lineage and non-neural cell lineage were used to investigate the role of the transcription factor MEF2A/D in neuronal survival and development in vitro. MEF2A has been shown to be present in undifferentiated NSCs. MEF2D expression increases as neurons mature, but its increase was not as spectacular as that of MEF2A [59,76]. Although MEF2A is present in undifferentiated NSCs, its level of expression appears to be relatively heterogeneous [76]. As the cells differentiate, the mean level of MEF2A expression was significantly higher in Tuj1-positive (Neuron-Specific Class III Beta-Tubulin) cells than in adjacent Tuj1-negative cells. Tuj1 is expressed only in neurons of the peripheral and central nervous system. It contributes to microtubule stability in neuronal cell bodies and axons and plays a role in axonal transport. These results suggest that MEF2A expression is increased in neuronal cells compared to non-neuronal. Moreover, shRNA-mediated knockdown of MEF2A reduced the number of neurons differentiated from NSCs compared to non-neural cells after their differentiation. Taken together, these data indicate that MEF2A is involved in the differentiation/maturation of neurons with NSCs. However, they do not exclude the possibility that MEF2A may be important for the development or survival of Tuj1-positive neurons after differentiation. Another published study indicates that the inhibition of MEF2 in cultured cortical neurons leads to apoptotic cell death suggesting that MEF2 is essential for neuronal survival, particularly newly differentiated neurons [61,77].

The importance of MEF2 transcription factors has also been demonstrated in vivo. Brain-specific triple knockout of MEF2A/C/D in mice has early postnatal lethality with increased signs of neuronal apoptosis [61]. Interestingly, single or double knockout mice did not show similar defects indicating a redundant role of MEF2 factors in neuronal survival. In a cerebral ischemia/reperfusion model, MEF2D silencing aggravated the neuroinflammatory response and brain injury, while its overexpression inhibited microglia activation, reduced cytokine levels and enhanced the viability of neurons exposed to oxygen–glucose deprivation and reoxygenation [78]. Recent evidence indicates that MEF2D may be of paramount importance for novel prosurvival strategies in some neurological diseases [79,80].

## 5. MEF2 in Neuronal Degeneration

### 5.1. MEF2A and MEF2D

Parkinson’s disease (PD) is characterized by the progressive and selective loss of dopaminergic neurons in the substantia nigra pars compacta (SNpc) and is the second most common neurodegenerative disease. Immunoblotting analysis of postmortem brain samples of PD patients revealed that the mitochondrial MEF2D level was preferentially reduced in these samples compared with matched controls, correlating closely with a downregulation of the ND6 protein [81]. Interestingly, Yang and coworkers demonstrated the MEF2D level to be increased in the brains of PD patients when compared to controls with a substantial portion of the protein localized to the neuronal cytoplasm. This correlated well with the high level of α-synuclein in PD brains [55]. In addition, the oxidized form of MEF2D was increased in the brain of postmortem PD patients [54]. MEF2D increases IL-10 microglia synthesis and decreases TNF-α, which negatively regulates inflammation and inflammation-induced cytotoxicity, which is an important aspect of PD [82]. MEF2D is also linked to Parkinson’s disease through the cyclin-dependent kinase 5 (Cdk5), which directly phosphorylates MEF2D at Ser444 [83]. Phosphorylation of MEF2D by Cdk5 leads to the inhibition of MEF2D transcriptional activity. Further studies have shown that phosphorylation of MEF2D at Ser444 renders MEF2D susceptible to specific degradation by caspases [84]. This diminished MEF2D transcriptional activity and attenuated the effect of MEF2D on neuronal survival. In neuronal cultures, MEF2D inhibition correlated with Cdk5-dependent neuronal death following exposure to stress conditions. Previous studies have shown that Cdk5 and its regulator, p35, colocalize in Lewy bodies [84,85].

Excitotoxic changes induced by quinolinic acid in the striatum have been found to induce apoptosis of dopaminergic (DA) neurons [86]. This correlated well with increased Cdk5 expression in apoptotic neurons. Subsequently, Cdk5 was shown to mediate 1-methyl-4-phenyl-1,2,3,6-tetrahydropyridine (MPTP)-induced death of DA neurons in the mouse PD model [87]. Recently, the relationship between Cdk5, MEF2D and loss of DA neurons has been established in the MPTP PD model [88]. The role of MEF2 in PD may also be related to the function of the dopaminergic system. Inhibition of MEF2D increased the toxicity of MPTP, a neurotoxin that induces degeneration of dopaminergic neurons producing persistent PD symptoms [89]. MPTP-mediated dopaminergic neuron loss has been shown to be dependent on the Cdk5 activator p35. Treatment with MPTP in vivo led to the phosphorylation of MEF2D at Ser444. The MEF2D mutant resistant to Cdk5 phosphorylation protected DA neurons from MPTP-induced toxicity in mice. Thus, modulation of MEF2D via Cdk5 is responsible, at least in part, for the MPTP-induced loss of substantia nigra dopaminergic neurons in vivo [90]. Recently, Cao and coworkers showed that enhancement of MEF2D by the naturally found glycoside, polydatin, protected the substantia nigra dopaminergic from degeneration in the MPTP model via a mechanism of glycogen synthase 3β (GSK-3β) inhibition [79]. Similarly, rhynchophylline, a major tetracyclic oxindole alkaloid in *Uncaria rhynchophylla* inhibited MPTP-triggered neurotoxicity by stimulating MEF2D via phosphoinositide 3-kinase/Akt/ GSK-3β signaling [91]. The neuroprotection in PD models involving modulation of MEF2D activity was also revealed for the following: chrysin [92]; T-006, a novel tetramethylpyrazine derivative [80]; salidroside [93]; the anti-cancer drug SU4312 [15]; a modified p5 peptide, TFP5 [94]; and danshensu, an active ingredient of *Salvia miltiorrhiza* [95]. The importance of MEF2D for neuronal protection was also demonstrated in a 6-hydroxydopamine (6-OHDA)-induced model of PD [14]. Injections of 6-OHDA to the SNpc region of the mouse brain induced miR-421 expression that negatively regulated MEF2D and disturbed the Bcl2/Bax ratio promoting cell death. In this model of PD, indirubin 3-oxime, a synthetic derivative of indirubin, prevented 6-OHDA-induced neurotoxicity by MEF2D activation via a mechanism potentially involving the inhibition of GSK-3β [96]. A growing body of evidence indicates the importance of macroautophagy in PD. However, relatively little is known about the role of chaperone-mediated autophagy (CMA) in the pathogenic process. A recent study demonstrated a link between CMA and MEF2D [55]. MEF2D interacts with CMA regulator Hsc70 through its N-terminal domain and is transported to lysosomes for degradation. Inhibition of CMA function led to accumulation of MEF2D in the cytoplasm, while increased CMA activity decreased it. Thus, MEF2D is considered as a CMA substrate. Maintaining adequate CMA activity is important for MEF2D function as the inhibition of lysosomal function reduced MEF2-dependent transcriptional activity [63]. Degradation of MEF2D via CMA can be interrupted by α-synuclein [63]. Overexpression of both wild-type and α-synuclein mutants has been shown to decrease binding between Hsc70 and MEF2D, cause accumulation of MEF2D in the cytoplasm and inhibit MEF2 activity. These results suggest that α-synuclein interferes with the normal turnover of CMA-dependent survival and MEF2D may be responsible for its toxicity and PD pathogenesis [55]. Both the wild-type and A53T mutant of α-synuclein were found to dysregulate the function of MEF2D in vitro. However, only the A53T mutant overexpressed in dopaminergic neurons was able to prolong HDAC4 nuclear retention and repress MEF2A leading to neuronal apoptosis in a MPTP PD model [97]. The dimers such as bis(3)-Cognitin (B3C) protected dopaminergic neurons from α-synuclein-induced cytotoxicity by enhancing MEF2D (but not MEF2A) activity in dopaminergic neurons with the basal conditions [98] suggesting that B3C may also restore MEF2 activity following neurotoxic insults. More importantly, B3C was able to ameliorate behavioral deficits in a mouse model of PD restoring MEF2D dysfunctions [98,99]. Human wild-type amyloid precursor protein (hAPP) can phosphorylate MEF2 by activating mitogen-activated protein kinase p38 (p38MAPK), which is a known modulator in AD. MEF2 may potentiate the antiapoptotic effect of hAPP in neurons [100,101].

Alzheimer-related dementia varies in severity, from mild cognitive impairment to fully developed Alzheimer’s disease (AD), which is characterized by the formation of β-amyloid plaques (Aβ) in neurons. Genetic analysis of MEF2A indicated a correlation between the Pro279Leu mutation in exon 8 of MEF2 and late AD suggesting that MEF2 is involved in AD pathology [102]. Polymorphisms of certain genes increase the risk for the rapid development of advanced Alzheimer’s disease (LOAD). LOAD is likely caused by a combination of acquired/environmental and genetic/inherited risk factors. Among genes involved in the development of LOAD are those that encode proteins regulating neurodegeneration and survival. The extended analysis of MEF2A genes in a total of 357 patients showed a Pro279Leu mutation in exon 8 and the polymorphism of polyglutamine (CAG) in exon 12 of MEF2A. These results suggest that the MEF2A gene may be associated with a risk of developing LOAD. Since MEF2 is linked with neuronal survival and the 279L allele was associated with decreased MEF2A transcriptional activity, the effect of this allele may be mediated by the reduction of antiapoptotic genes [102]. A recent study identified MEF2A as the cysteine histidine-rich protein (PINCH) transcription factor in neuroinflammation [103]. PINCH is barely detectable in healthy neurons, but its expression becomes evident in tauopathy-associated human diseases, such as AD [104]. Activation of MEF2A via Cav3.1-dependent Ca^2+^ entry upregulated PINCH expression leading to the sequence of events destabilizing the kinesin-dependent mitochondrial machinery and neuronal metabolism [103]. Alzheimer’s disease is also characterized by downregulation of autophagy-related-genes that are regulated by MEF2A. Methylated-microarray analysis performed by Li and colleagues [105] showed that methylation level of the enhancer region of MEF2A was increased in AD. It can be speculated that the increased methylation reduces the expression of MEF2A and, as a consequence, downregulates autophagy-related genes that are closely linked to AD pathology. MEF2D in the context of AD has not been extensively studied. One report demonstrated that protocatechuic acid promoted the expression of MEF2D and suppressed GSK-3β in an okadaic acid-induced AD model, attenuating autophagy via regulation of the Akt/ GSK-3β/MEF2D pathway [106]. In APP/PS1 double transgenic mice expressing a chimeric mouse/human amyloid precursor protein and a mutant human presenilin 1, galangin upregulated MEF2D and Akt and improved pathological changes in the hippocampus suggesting that neuronal injury in this brain structure may be related to the Akt/MEF2D/Beclin-1 signaling pathway [107]. The function of MEF2 in retinal ganglion cell (RGC) survival was studied in MEF2 knockout mice. RGCs degeneration is responsible for vision loss in glaucoma, a disease affecting nearly 80 million people worldwide with 10% expected to lose vision completely [108,109]. Other ophthalmic conditions leading to RGC death include ischemic optic neuropathy, optic nerve inflammation and optic nerve injuries. Deletion of all MEF2A, MEF2C and MEF2D alleles had no effect on RGC survival during development but had a positive effect on the survival of mature RGCs. The data suggests that most of the neuroprotective effect was achieved by complete deletion of MEF2A. Knockout of MEF2D was not sufficient to produce similar change, although increased the positive effect of MEF2A knockout. Conversely, overexpression of MEF2A in wild-type mice reduced survival of RGC after optic nerve crush [110,111]. MEF2A and MEF2D are the major isoforms expressed in RGCs [83,100]. Although MEF2D was not required for RGC development, global MEF2D knockout caused selective postnatal photoreceptor cell degeneration and vision loss [112,113]. MEF2D function in developing photoreceptor cells has been attributed, inter alia, to the absence of other MEF2 isoforms in this cell type and coregulation of photoreceptor genes via Cone-Rod Homeobox (CRX) transcription factor. MEF2 is usually considered neuroprotective in cellular stress models. On the other hand, MEF2A gene silencing has been shown to prevent RGC from apoptosis and improves survival after optic nerve crush [111].

### 5.2. MEF2B

The pattern of MEF2B expression indicates that MEF2B, like its paralogs, may contribute to development and maintenance of a variety of tissue types. However, mice null for MEF2B were viable and had no obvious abnormalities indicating that MEF2B does not contribute to the development or other MEF2 proteins are able to compensate for MEF2B loss during development [114]. So far, the only noncancer disease in which MEF2B may be involved in is intellectual disability. This correlation is, however, based on rather weak evidence showing codeletion of MEF2B together with 10 or 75 other genes in two studied patients with diagnosed intellectual deficits [115].

### 5.3. MEF2C

Among MEF2 proteins, MEF2C is the one associated with the widest range of neuropsychiatric diseases (Table 2). This probably arises from the ability of MEF2C to regulate the expression of more than a thousand genes in the developing brain that regulate neuronal migration [116], differentiation [76,116,117], axon guidance and pruning [69] and axonal remodeling [118,119]. The intensive studies of human genome and genome sequencing of a large number of patients demonstrated that MEF2C should be considered as a candidate risk gene in a variety of neuropsychiatric disorders, including Alzheimer’s disease (AD) [120], autism spectrum disorders [121], schizophrenia [122], bipolar disorder [123], Angelman syndrome [124], major depression [125], attention deficit and hyperactivity disorder [126], epilepsy [127] and Parkinson’s disease [128]. Although most of these studies observed a disease-associated single nucleotide polymorphism, they collectively highlight the role of MEF2C in the proper function of the human brain. Several groups [129,130,131,132] also reported microdeletions or missense or nonsense mutations in the open reading frame of the MEF2C gene that are linked to severe neurodevelopmental disease, named MEF2C Haploinsufficiency Syndrome (MCHS). This relatively new disorder described in 2009 [129,133] is characterized by variable degrees of developmental delay, severely impaired or absent speech, autistic and stereotypic behavior, hypotonia, seizures, ametropia, hemangiomas and characteristic facial features. A recent study on DNA binding-deficient Mef2c^+/−^ mice showed that MCHS-associated missense mutations cluster in the conserved DNA binding domain of MEF2C may be responsible for autism-related behaviors, changes in the cortical gene expression and deficits in cortical excitatory synaptic transmission [134]. Moreover, Tu and colleagues [135] reported that these mice presented profound changes in gene expression associated with neurogenesis, synaptic formation and neuronal apoptosis. Accordingly, Mef2c^+/−^ animals had decreased neurogenesis and enhanced neuronal death as well as an altered ratio of excitatory to inhibitory hippocampal neurotransmission. The same authors also demonstrated that neurobehavioral deficits and histological damage may be mitigated by a chronic treatment with nitrosynapsin, a memantine-related uncompetitive inhibitor of NMDA receptors.

A recent review by Perea and colleagues [136] discussed how microglia contributes to AD pathology by secreting various neurotoxic agents. Mice lacking MEF2C in microglia presented an exaggerated microglial response and suppressed social behaviors when challenged with interferon type I (IFN-I) [57]. Moreover, the siRNA-mediated silencing of MEF2C in primary microglia combined with lipopolysaccharide stimulation increased production of proinflammatory cytokines and chemokines, which are also overexpressed in aged microglia [57]. Therefore, loss of MEF2C function may participate in the development of proinflammatory milieu in the AD brain and negatively affect cognitive function exacerbating disease pathology. The importance of MEF2C in microglia is further emphasized by the recent study of Xue and colleagues [137]. They found that MEF2C nuclear translocation was inhibited in a mouse model of brain amyloidosis. In this model, oligomeric amyloid-β42 affected MEF2C deregulation and enhanced microglial activation, which can be reversed by blocking IFN-I signaling. MEF2C has also been found to regulate APP processing [138] and participate in amyloid protein precursor (APP)-dependent neuroprotection [101].

High expression of MEF2C in brain regions traditionally linked with learning and memory [139] is another presumption for its contribution to cognitive impairments in AD. A large meta-analysis conducted in 2013 on 74,046 individuals [140] has confirmed that the rs190982 polymorphism in MEF2C on chromosome 5 can be a protective factor against AD dementia in the Caucasian population. The association between rs190982 polymorphism in the MEF2C gene and AD has also been documented in a large Spanish population [141]. Contrary to this, a larger genome-wide association meta-analysis performed on clinically diagnosed AD samples in 2019 was unable to reproduce these findings [142]. The study of Jansen and colleagues was also unable to replicate the association between the MEF2C polymorphism and AD [143]. Another two studies also failed to reveal any associations between the rs190982 polymorphism in the MEF2C gene with AD susceptibility in a Han Chinese cohort [144,145]. These distinct results may be attributed to genetic heterogeneity between selected ethnic cohorts, which does not exclude genome-wide association studies as a valuable tool for identification of candidates for AD-related genetic variants.

The involvement of MEF2C has also been reported for epilepsy. A literature review performed by Borlot and coworkers [146] showed that 19 out of 23 analyzed patients harboring MEF2C pathogenic variants or microdeletions had seizures reported in the first 12 months of life. Similar observations were made by a retrospective study on 16 patients with MEF2C haploinsufficiency [147]. Moreover, subjects with partial MEF2C deletion have a lower risk for developing epilepsy compared to those with complete loss of MEF2C function. The genetic screening of patients with different forms of encephalopathy revealed several mutations in the MEF2C gene that may be potentially associated with seizure generation. So far, this includes: two missense mutations, p.G27A and L138Q [130]; three truncating mutations p.E34X, p.H76DfsX15 [130] and p.S228X [132]; the de novo frameshift mutation c.457delA [148]; and few associated with 5q143 microdeletions [149]. Novel point mutations have recently been described in a Chinese population [150]. The MEF2C target gene expression in epilepsy may also be affected by other pathogenic mutations. Xu and coworkers in their newest case report identified a novel mutation in salt-inducible kinase 1 (c.880G  > A chr21: 43,420,326 p. A294T) in a patient with epileptic encephalopathy-30. This mutation dysregulated cellular transcriptome and metabolome, specifically MEF2C-dependnet genes and certain epilepsy causing genes [151]. In post-traumatic epilepsy (PTE), the expression of MEF2C was significantly upregulated, which negatively correlated with the expression of miR-30e-5p and miR-98-5p [152]. Combined with the massive Ca^2+^ influx after traumatic brain injury, MEF2C may promote NMDA receptor expression, potentiating Ca^2+^ influx, which is one of the main triggers in the occurrence of PTE. Similarly, miR-30e-5p and miR-98-5p may contribute to the development of PTE by affecting MEF2C expression. As MEF2C participates in activity-dependent synaptic elimination, any dysfunctions in MEF2C may restrict this process leading to autism spectrum disorders (ASD) syndrome. The studies on MEF2C knockout mice demonstrated an increased number of excitatory synapses and potentiated synaptic transmission in the hippocampus, producing learning and memory impairments and behavioral deficits similar to ASD [9]. Interestingly, the postnatal loss of MEF2C did not result in altered memory, long-term potentiation or social behaviors [8] suggesting that regulation of these functions in ASD may depend on MEF2C expression during brain development. ASD has a strong genetic underpinning and there is a significant overlap between MEF2C-regulated genes and candidate autism risk genes [62], perhaps explaining social and behavioral deficits in MEF2C mutant mice. The presence of autism-related symptoms has also been observed in MCHS. Of four patients described by Vidal and colleagues [153] who manifested severe intellectual disability with autistic feature, three of them presented behavior characteristic for MCHS. MEF2C is also linked to Mecp2 [130,154] and Ube3a [124], which are involved in Rett and Angelman syndromes, respectively. Rett syndrome is a severe neurodevelopmental disorder that almost exclusively affects women and is caused by mutations in the Mecp2 gene. In patients with MCHS, the expression of Mecp2 was decreased and MEF2C has been shown to activate the Mecp2 promoter [130]. MEF2C knockout mice displayed a spectrum of behavioral phenotypes that were observed in a Rett syndrome (RTT) animal model. This includes the stereotypy as well as morphological alterations resembling those observed in RTT models [155]. Based on these observations, it has been concluded that RTT or RTT-like phenotypes arise from a similar origin involving defects in a common signaling pathway driven by MECP2 and MEF2C. In line with that, Wang and colleagues [155] identified MEF2C point pathogenic mutations in the absence of Mecp2 gene mutations in patients with RTT suggesting that the MEF2C gene itself can be considered a candidate for a risk gene in RTT. The involvement of MEF2C in the pathogenesis of ASD has been recently discussed by Chaudhary and coworkers [156]. Two reports showing the potential involvement of MEF2C in ASD have been recently released (2022). Matrisciano and coworkers demonstrated changes in the expression of genes involved in excitatory and inhibitory neurotransmission including MEF2C in a mouse model of idiopathic autism [157]. DNA binding-deficient global MEF2C heterozygous mice (MEF2C-Het), which is a commonly used model of ASD, exhibited functional impairments of peripheral auditory nerve and a modest reduction in hearing sensitivity [158]. Deficiency in MEF2C was associated with multiple cellular alterations, including abnormal myelination of the auditory nerve, mitochondrial dysfunctions and increased cochlear inflammation. The information available in the Psychiatric Genome Association reported MEF2C motif enrichment in sequences surrounding the top scoring single nucleotide polymorphism within risk loci related to schizophrenia heritability [122]. A genome-wide association study demonstrated that among 349 genes in 108 schizophrenia-associated loci, the MEF2C gene was one of only few to be overexpressed preferentially during the middle-to-late stage of cortical development [159].

**Table 2:** Effect of MEF2C genetic manipulations on neuronal and behavioral features.

	Neuronal Effect	Behavioral Effect	References
Conditional knock-out of MEF2C in Calcium/calmodulin-dependent protein kinase II (CaMKII)- Cre93 line after birth.	Increased quantity of spines in the mice hippocampus.	No changes in learning and memory ability, LTP or social conduct.	[160]
Removal of MEF2C in mice forebrain at late embryonic day. Overexpression of MEF2C in transgenic mice (NSE-MEF2C-VP16 mice).	Increased quantity of spinous processes and excitatory synapses. Intensified basal and induces synaptic transmission. Decrease in glutamatergic synapse density of hippocampal pyramidal neurons.	Dependent on hippocampus learning and memory diminished ability.	[9]
Silencing of MEF2C.	Condensation of branches and spines on dendrites of Purkinje cells and impaired localization of synaptic proteins.		[116,117]
Conditional knock-out of MEF2C in the progenitor/stem cells.	Neurons from cortical plate with atypical cell body size and density.	Strong changes in behavior and electrophysiological features typical for immature neurons and severe behavioral deficits reminiscent of Rett syndrome, an autism-related disorder.	[116]
Knockdown of MEF2C in vivo and in vitro.	Suppression of nicotine-mediated modifications of dendritic complexity.	Diminished nicotine-dependent changes in passive avoidance behavior.	[161]
Knockdown of MEF2C in excitatory neurons of embryonic mice cortex and hippocampus (EmxCre × MEF2C flox/flox).	Reduction of cortical network activity due to increase in inhibitory and decrease in excitatory synaptic transmission. MEF2C regulates inhibitory/excitatory (E/I) synapse density.	Deficiency in fear learning, memory, various social conduct, socially-motivated ultrasonic vocalizations and reward-related behaviors, features reminiscent of autism, intellectual disability and schizophrenia.	[62]
Silencing of MEF2C by HSV-Cre-GFP virus in MEF2C fox/fox mice at postnatal day 2 (P2) or at postnatal day 14–15 (P14–15). Overexpression of MEF2C by electroporation of pcBIG-MEF2C-VP16 plasmids at E12.5 in wild-type embryos.	Amplified number of dendritic spines in striatal projector neurons (SPNs) at P8. Reduced number of dendritic spines in SPNs at P14.	Malfunctioning neonatal isolation-induced USVs (form of vocal communication in neonatal rodents).	[162]
Knockdown of postnatal MEF2C AAV-Cre-GFP neocortical cultures.	Reduction of pyramidal neurons excitatory synapses. Decreased spine density on normal branching dendrites in neurons.		[163]
Exon 2-deleted allele of MEF2C.	Lower neurons number and axon length. Impaired dendritic connections. Elevated E/I ratio hippocampus.	Autism-like symptoms, intellectual disability, speech deficiency, seizures or motor impairments.	[135]
MEF2C overexpression in mature prefrontal cortex (PFC) by AAV-MEF2C virus.	Reduced mushroom spines ratio in layer III of PFC and no alteration in total spine quantity.	Congenital improvement.	[122]

The data derived by the Schizophrenia Working Group of the Psychiatric Genomics from the set of 108 schizophrenia risk haplotypes identified at least two motifs related to the potential MEF2C target sites [164]. The same study found that MEF2C binding motifs were significantly overrepresented among nearly 1000 nucleosomal sequences affected by histone H3K4 hypermethylation in the diseased cohort. MEF2C downregulation in cell culture models produced abnormal H3K4 methylation mimicking the changes reported in the prefrontal cortex of schizophrenic individuals [122]. On the other hand, adeno-associated virus-mediated MEF2C upregulation in mouse prefrontal cortical projections enhanced working memory and object recognition abilities in conjunction with local synaptic remodeling [122]. Therefore, MEF2C should be considered as a promising therapeutic target for the treatment of schizophrenia-related cognitive deficits.

## 6. Conclusions

The transcription factors of the MEF2 family play a critical role in neuronal development by regulating the expression of hundreds of genes during precisely defined phases of brain morphogenesis and subsequent neuronal maturation, differentiation and survival. Since its initial discovery as a key transcriptional regulator in skeletal muscles, it became apparent that MEF2 has a profound effect on neuropsychiatric phenotypes. The exact mechanisms of how MEF2 is linked to numerous mental illnesses are not fully understood yet. Current studies continuously identify novel mutations or deletions in MEF2 genes in neurological diseases, but the data are still too limited to make a conclusion about their influence on healthy brain development or disease origin. We have only begun to understand the function of MEF2A and MEF2D in neurons, and the MEF2B that is also expressed in the central nervous system remains a mystery. Additionally, the contribution of MEF2 to the physiology of other brain cells, such as astrocytes or microglia, should also be included in the mainstream of research on MEF2. It is also noteworthy that MEF2 should become considered as a more attractive potential target in the treatment of many diseases. Although previous studies demonstrated various functions of MEF2, and sometimes contradictory findings, further research is still needed to elucidate the pathological mechanisms by which MEF2s are linked to defective signaling pathways. Undoubtedly, thorough studies of these mechanisms and their downstream effects would facilitate and improve therapeutic strategies in neuropsychiatric diseases.

## Figures and Tables

**Table 1:** MEF2 transcription factor family gene expression in developing mouse brain (adapted from [56]).

		MEF2A	MEF2B	MEF2C	MEF2D
**Olfactory bulb**	E16.5	+	+	++	++
	neonatal	+	++	+++	++
	adult	+	+++	+++	++
**Amygdala**	E16.5	−	+/−	+++	++
	neonatal	−	−	+	+
	adult	+	−	++	++
**Cortex**	E16.5	+	++	+++	++
	neonatal	+	+++	+++	++
	adult	+	+++	+++	++
**Hippocampus**	E16.5	−	+	+++	+
	neonatal	+++	+	++	+
	adult	+++	+/−	++	+++
**Thalamus**	E16.5	+++	+	+++	+
	neonatal	+++	+	+	+
	adult	+++	−	++	++
**Midbrain**	E16.5	+	−	++	++
	neonatal	+	+	++	++
	adult	−	++	+++	++
**Cerebellum**	E16.5	−	−	++	++
	neonatal	+	++	++	+
	adult	+	−	+	++
**Pontine nuclei**	E16.5	++	+/−	++	+
	neonatal	++	+	++	+
	adult	+	++	+++	++
**Medulla**	E16.5	+	+	+	+
	neonatal	+	+	+	+
	adult	nd	nd	nd	nd

+ low expression, ++ moderate expression, +++ high expression, +/− expression barely detectable, −no expression, nd—not determined.
